# Circulating Tumor DNA in Breast Cancer: Diagnostic Insights From a Case Series

**DOI:** 10.7759/cureus.100833

**Published:** 2026-01-05

**Authors:** Cortney McKay, Tasnim Rahman, Bipin Ghimire, Marian Girgis

**Affiliations:** 1 Hematology and Medical Oncology, Henry Ford Health System, Detroit, USA; 2 Internal Medicine, Henry Ford Health System, Detroit, USA

**Keywords:** breast cancer, breast cancer biology, circulating tumor dna (ctdna), guardant360, liquid biopsy, medical oncology, minimal residual disease, molecularly targeted therapy, signatera ctdna

## Abstract

Circulating tumor DNA (ctDNA), the tumor-derived fraction of cell-free DNA, has emerged as a valuable biomarker for monitoring treatment response, detecting minimal residual disease (MRD), and identifying early cancer recurrence. While histologic tissue diagnosis remains the gold standard for confirming malignancy, guidelines from organizations such as the National Comprehensive Cancer Network (NCCN) and the European Society for Medical Oncology (ESMO) acknowledge that ctDNA may serve as a supplemental tool in rare instances where tissue is unobtainable. In such cases, results should be interpreted alongside clinical and radiologic findings, with tissue confirmation pursued whenever possible. This case series presents three distinct breast cancer cases in which ctDNA-based MRD detection was instrumental in identifying recurrence and guiding precision therapy based on actionable genomic alterations when biopsy was not feasible or inconclusive.

## Introduction

Cell-free DNA (cfDNA) comprises short DNA fragments released into the bloodstream during apoptosis of both normal and malignant cells. Circulating tumor DNA (ctDNA) is a tumor-specific subset of cfDNA, distinguished by unique genomic alterations, such as somatic mutations, that are absent in cfDNA of non-tumor origin. Increasingly, ctDNA is being studied across multiple malignancies for its role in tumor molecular profiling, monitoring therapeutic response, detecting treatment resistance, enabling early cancer detection, and identifying post-treatment MRD [1,2,3].

In breast cancer, particularly in high-risk settings, retrospective studies suggest that ctDNA-based MRD detection can facilitate earlier recognition of disease recurrence. Previous studies have shown that ctDNA can detect micro-metastatic relapse before clinical or radiologic evidence is apparent, enabling prompt diagnostic work-up and diagnosis [4].

Up to 30% of breast cancer survivors, and more than 40% of those with stage I-III triple-negative breast cancer (TNBC), experience disease recurrence following standard therapy, emphasizing the need for improved early detection strategies. ctDNA-based MRD detection has demonstrated the ability to predict recurrence risk, inform treatment decisions, and improve clinical outcomes [5].

Recent clinical trials highlight the potential of ctDNA to guide real-time cancer management. In the phase III SERENA-6 trial of HR-positive/HER2-negative metastatic breast cancer, serial ctDNA monitoring for emerging ESR1 mutations enabled therapy modification prior to radiographic progression, resulting in prolonged progression-free survival and delayed quality-of-life deterioration [6]. Similarly, the randomized DYNAMIC trial in stage II colon cancer showed that using ctDNA to guide adjuvant therapy safely reduced the need for chemotherapy without worsening recurrence-free or overall survival [7]. Together, these findings highlight ctDNA’s potential to guide timely treatment escalation, though initiating therapy based solely on ctDNA without tissue confirmation is not yet standard practice [4].

This case series presents three distinctive cases of breast cancer recurrence in which ctDNA-based MRD detection was instrumental in distinguishing between malignancy types and guiding early treatment decisions using actionable alterations when tissue biopsy was not feasible or inconclusive.

## Case presentation

Case 1

The first case is a 64-year-old female with a history of bilateral invasive ductal carcinoma of the breast diagnosed in 2016. She underwent a bilateral modified radical mastectomy with a sentinel lymph node biopsy. Surgical pathology revealed a 1 cm tumor in the right breast with negative lymph nodes, and a 2.2 cm tumor in the left breast with two out of eight positive lymph nodes. Immunohistochemistry revealed estrogen receptor (ER) positive, progesterone receptor (PR) positive, and human epidermal growth factor receptor 2 (HER2) negative status. She received adjuvant radiation to the left chest wall and axillary nodes. Although adjuvant chemotherapy was the standard of care then, she declined it due to low Oncotype DX scores of 10 and 9 for the right and left breasts, respectively. She began adjuvant endocrine therapy with tamoxifen, later transitioning to anastrozole after achieving menopause.

Approximately six years later, she developed a gradually enlarging neck mass. A biopsy performed two years earlier had been read as benign. With continued growth, she underwent an excisional biopsy and cervical lymph node dissection. Pathology revealed a 6 cm high-grade undifferentiated spindle cell sarcoma. Staging scans showed no metastatic disease. She received four cycles of adjuvant Adriamycin, ifosfamide, and Mesna (AIM).

Around 1.5 years after completing AIM, a surveillance PET-CT showed an FDG-avid lesion in the right adrenal gland and retrocaval lymph node. A follow-up CT adrenal protocol showed progressive asymmetric thickening of the right adrenal gland, suspicious for an adenoma versus metastasis. Due to the small size and location of the lesions, neither a CT-guided biopsy nor a surgical biopsy was feasible or safe, respectively.

Given her history of multiple malignancies, a tumor-informed ctDNA assay (NeXT Personal platform) was performed. Archival tumor tissue from her prior breast cancer and sarcoma was analyzed. ctDNA was negative for sarcoma but positive for breast cancer, with a quantified level of 2,175 parts per million (PPM). These findings raised concern for possible metastatic breast cancer.

**Figure 1:**
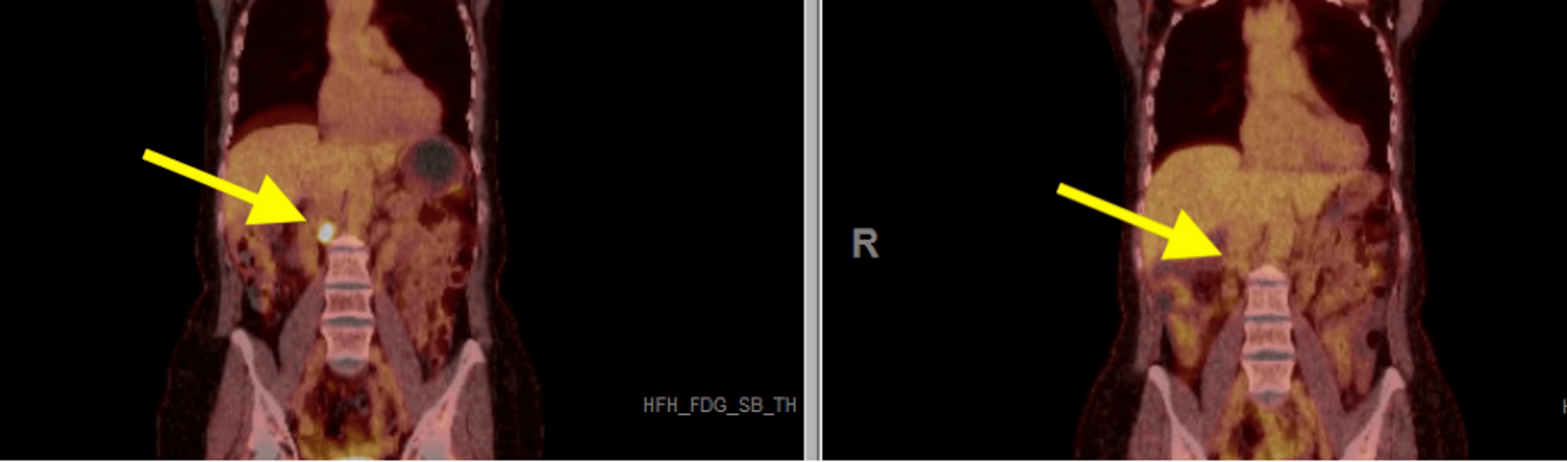
Comparison of PET-CT scans over six months of systemic therapy demonstrates near-complete resolution of the metastatic right adrenal lesion. PET-CT: Positron Emission Tomography Computed Tomography

Follow-up imaging demonstrated progression involving the lymph node and right adrenal gland. An adrenal biopsy confirmed metastatic carcinoma consistent with a breast primary. Blood-based next-generation sequencing (NGS) identified a PIK3CA mutation. She began systemic therapy with ribociclib, inavolisib, and fulvestrant. Six months later, PET/CT showed near-complete resolution (Figure 1). Additionally, tumor-informed ctDNA testing was negative for detectable ctDNA.

Case 2

The second case involves a 70-year-old postmenopausal woman who underwent screening mammography in December 2020, which showed architectural distortion in the upper outer quadrant of the left breast. Biopsy revealed invasive mammary carcinoma with mixed ductal and lobular features. Immunohistochemistry was positive for ER and PR and negative for HER2. A breast MRI identified two prominent axillary lymph nodes. She subsequently underwent a left modified radical mastectomy with axillary lymph node dissection. Final pathology showed a 9 cm grade 2 invasive lobular carcinoma with five out of seven lymph nodes positive for metastasis, indicating a final prognostic stage of IB (pT3aN2a). She went on to receive adjuvant radiation to the chest wall and axilla. She then began adjuvant anastrozole but discontinued it after five months due to persistent arthralgias. She did not receive a CDK4/6 inhibitor due to financial constraints.

**Figure 2:**
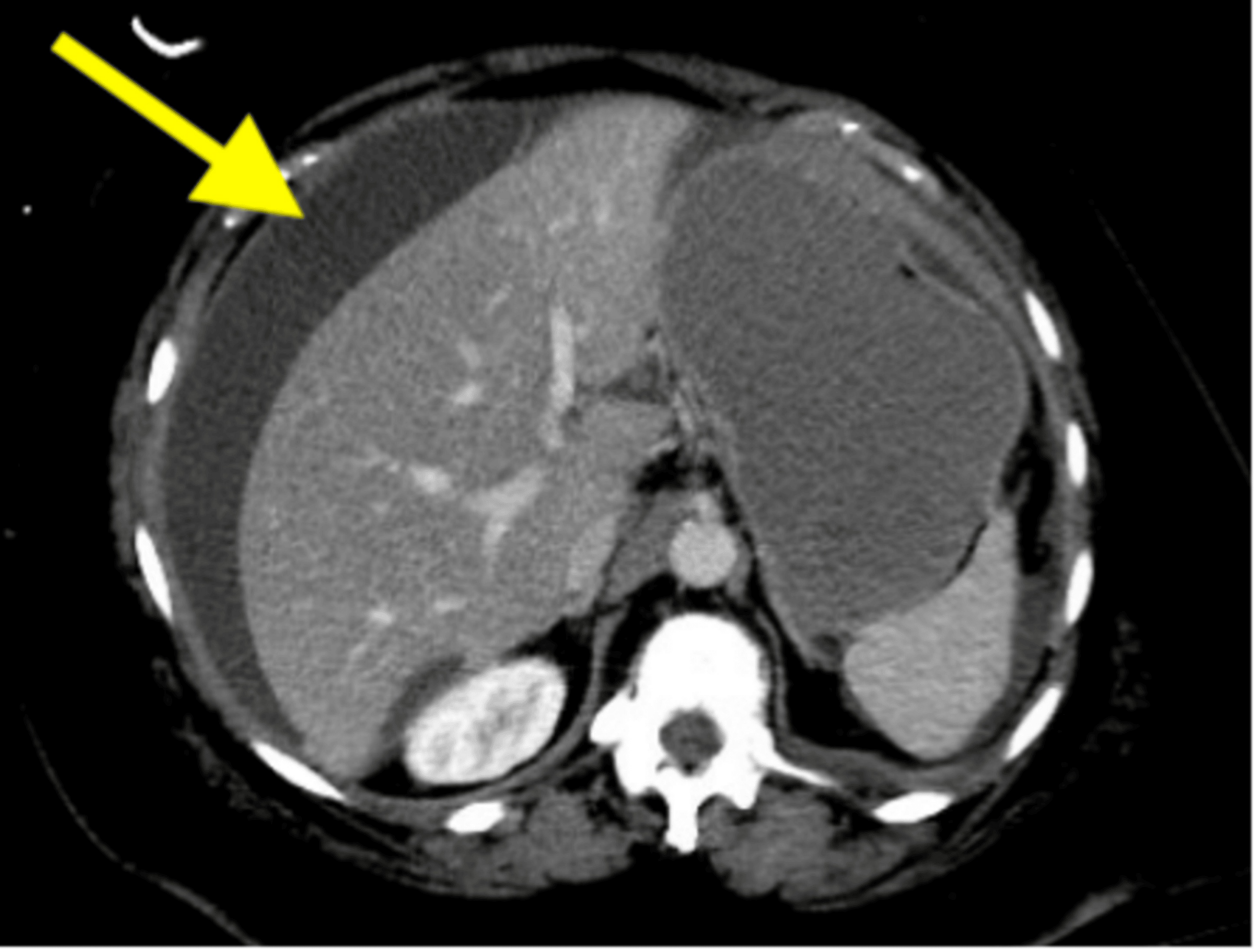
Axial cut of CT abdomen demonstrating a large volume of ascites with thickened and minimally nodular peritoneal enhancement, including thickening of the subdiaphragmatic left hemidiaphragm upwards to 13 mm.

She remained in remission until December 2024, nearly three years after discontinuing adjuvant therapy, when she was admitted to the hospital for abdominal distension and fatigue. CT of the chest, abdomen, and pelvis revealed bilateral pleural effusions, malignant appearing ascites, diffuse sclerotic bone lesions, and peritoneal carcinomatosis (Figure 2). Despite multiple paracenteses, peritoneal cytology remained either negative or indeterminate. A left iliac bone biopsy showed no histologic evidence of malignancy. Interventional radiology concluded that the peritoneal lesions could not be safely biopsied.

Given her history and high clinical suspicion of recurrence, tumor-informed ctDNA testing was performed using the NeXT Personal platform on the original tumor tissue. ctDNA was positive at 1739 PPM. Tumor markers were also elevated with CA 15-3 level of 373.9 U/mL (normal <32.5), and CA 27.29 level of 739 U/mL (normal <38.6). Despite a lack of confirmatory histopathology, the clinical and molecular findings strongly supported a diagnosis of recurrent breast cancer. As a result, she was initiated on systemic therapy with ribociclib and anastrozole. Shortly after, the patient died from renal failure, so we could not ascertain her response.

Case 3

The final case is of a 49-year-old female who initially presented with right breast pain and nipple retraction. A diagnostic mammogram in September 2022 revealed distorted architecture in the right breast. Follow-up MRI breast a month later demonstrated a mass in the lower inner quadrant of the right breast with asymmetric, enlarged ipsilateral axillary lymph nodes. A core needle biopsy of the right breast mass was performed and revealed a grade 3 poorly differentiated invasive ductal carcinoma, with involvement of regional lymph nodes, indicating a clinical stage of IIA (T3N1M0). The tumor was ER low, PR negative, and HER2 negative. Follow-up staging CT chest, abdomen, and pelvis revealed pulmonary nodules; however, they were not feasible to biopsy.

As a result, she initiated neoadjuvant therapy with dose-dense doxorubicin, cyclophosphamide, and pembrolizumab, followed by four cycles of paclitaxel, carboplatin, and pembrolizumab, which she completed in April 2023. In May 2023, the patient underwent bilateral mastectomies with a right-sided targeted sentinel lymph node biopsy. Pathologic examination of the right breast revealed a separate 0.8 cm grade 3, ER strongly positive, PR moderately positive, HER2 negative primary invasive ductal carcinoma. Furthermore, the site of the previously biopsied tumor, as well as the sentinel lymph node biopsy, revealed a complete pathological response. Imaging also revealed resolution of the pulmonary nodules, supporting potential initial metastatic involvement.

Following surgery, she completed adjuvant radiation therapy, as well as pembrolizumab, in October 2023. Although the ER low breast cancer had a complete response to neoadjuvant treatment, she was started on capecitabine in August 2023, given concern for metastatic pulmonary nodules; however, this was discontinued after 6 months due to toxicity.

Since the final pathology revealed a second, distinct hormone receptor-positive primary tumor in the breast, she started adjuvant endocrine therapy with letrozole. To monitor for MRD, the patient was followed with Signatera, a personalized tumor-informed ctDNA assay.

**Figure 3:**
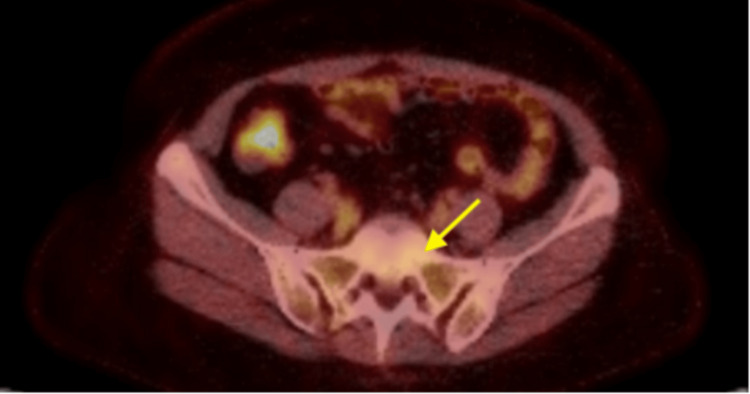
PET-CT scan demonstrating multiple small sclerotic foci within multilevel vertebral bodies and sacrum.

In March 2025, Signatera detected ctDNA consistent with residual disease from both tumor clones, prompting further evaluation with PET-CT imaging. The scan was suspicious for osseous metastases; however, the lesions were too small to biopsy (Figure 3). Liquid biopsy with Guardant360 was used to identify actionable mutations, including ESR1, given her previous exposure to letrozole. Testing revealed PIK3CA and TP53 alterations. Based on these findings, the patient was initiated on therapy with fulvestrant, ribociclib, and inavolisib, a PI3Kα inhibitor. Although the previous ER low clone was detected, it was carefully decided to start with a less toxic regimen while carefully monitoring both clones with Signatera and imaging. Repeat PET-CT 3 months after initiation of treatment showed stable osseous metastasis without progression of disease. Fortunately, repeat Signatera assays performed on both tumor clones at this time point demonstrated a significant reduction in ctDNA levels.

## Discussion

These three cases represent the role of ctDNA in modern oncology. In breast cancer, it is currently approved for molecular profiling to guide targeted therapy in the metastatic setting. Key actionable alterations detectable via ctDNA include ESR1, PIK3CA, AKT/PTEN mutations, microsatellite instability, and NTRK/BRAF mutations [4].

Emerging retrospective evidence suggests that detecting either tumor-informed or tumor-uninformed ctDNA during surveillance after definitive treatment for early-stage breast cancer may predict metastatic recurrence [4]. Nevertheless, the current standard of care continues to rely on imaging followed by biopsy to confirm recurrence, particularly in patients with a prolonged disease-free interval [8].

In all three cases, histologic confirmation was either not feasible or yielded inconclusive results due to clinical constraints. In Case 1, diagnostic complexity was heightened by the patient’s history of two distinct malignancies, breast cancer and sarcoma. Imaging findings initially raised concern for sarcoma recurrence, given its more recent activity; however, biopsy was precluded by the lesion’s size and location. In Case 2, the patient had remained in remission for over three years following breast cancer treatment before presenting with pleural effusions, ascites, osseous lesions, and peritoneal carcinomatosis. Despite two biopsy attempts, pathology remained nondiagnostic, creating a management conundrum. In Case 3, new osseous metastases raised suspicion for cancer recurrence, but the lesions could not be safely biopsied. As a result, Signatera testing was pursued to guide therapeutic decision-making. In all three cases, systemic treatment was initiated in the absence of definitive biopsy confirmation, informed instead by integrated clinical, radiologic, and molecular ctDNA findings.

Consequently, in the first two patients, we pursued ctDNA MRD analysis using a tumor-informed approach with the NeXT Personal platform, which utilizes whole-genome sequencing of both tumor and matched normal samples to identify up to 1,800 somatic variants unique to the tumor. A personalized panel targeting these variants is then used to analyze cfDNA from plasma with ultra-sensitive detection capability. Results are reported in PPM, representing the number of tumor-specific molecules detected per million cfDNA molecules sequenced. The assay has demonstrated 99.9% specificity for ctDNA detection [9].

In the first case, ctDNA was positive when the sequencing panel was derived from breast cancer tissue, with no detection using sarcoma-derived material. A subsequent biopsy confirmed breast cancer recurrence. While biopsy ultimately confirmed the diagnosis, earlier ctDNA detection allowed us to initiate shared decision-making discussions and prepare a treatment strategy in advance. We were also prepared to initiate systemic therapy based on ctDNA results alone, had biopsy remained unfeasible [9].

In the second case, systemic therapy was urgently initiated based on ctDNAs findings due to the patient’s disease burden and symptoms. The positive ctDNA results, aligned with rising breast tumor markers, supported initiation of treatment despite the lack of confirmatory histopathology.

The third case employed liquid biopsy methods, including Signatera® and Guardant360®, to analyze circulating tumor DNA (ctDNA). Signatera is a personalized, tumor-informed assay designed to detect minimal residual disease (MRD) and monitor for recurrence or treatment response following curative-intent therapy. Using whole-exome sequencing of tumor and matched normal DNA, a patient-specific genomic profile is created, from which 16 clonal single-nucleotide variants are selected to develop a custom multiplex PCR panel. At each testing interval, plasma cfDNA is extracted, amplified for these target mutations, and analyzed using ultra-deep NGS, enabling detection of ctDNA at variant allele frequencies as low as 0.01%. MRD positivity is defined as the detection of at least two of the tracked mutations above background error rates, allowing for highly sensitive, longitudinal surveillance. In this patient, who had a history of two distinct breast cancers, Signatera detected both tumor clones at the time of recurrence. While typically used in early-stage cancers to track mutations from the original tumor, in this case, it was specifically applied for MRD monitoring. In contrast, Guardant360 is a fixed-panel ctDNA assay that provides comprehensive genomic profiling across a broad set of cancer-related genes, enabling identification of targetable alterations when deciding on treatment therapy [10].

The third case is particularly noteworthy, as it highlights the clinical utility of ctDNA in the management of TNBC, a subtype known for its aggressive behavior and high risk of recurrence. This case illustrates how ctDNA analysis can play a dual role: first, through Signatera, by detecting MRD and identifying early molecular recurrence prior to radiographic evidence; and second, through Guardant360, by enabling precision oncology in situations where tissue biopsy is not feasible. In our case, the osseous metastatic lesions were too small to biopsy. Together, these tools not only enhance early detection and surveillance but also support informed treatment decisions in a clinically challenging setting.

ctDNA-guided cancer management is under active investigation across tumor types. Adjuvant therapy informed by ctDNA has already been reported in colorectal cancer, supported by the DYNAMIC trial [7]. Clinical trials in breast cancer are ongoing in both adjuvant and metastatic settings. Although more evidence is needed before ctDNA can replace tissue biopsy for detecting recurrence, our cases highlight its utility, especially when biopsy is infeasible or inconclusive despite high clinical suspicion.

## Conclusions

In conclusion, while ctDNA has established clinical utility in guiding early targeted therapy for metastatic breast cancer through molecular profiling, its role without biopsy confirmation remains investigational. Standard practice prioritizes imaging and biopsy for confirmation, but this case series highlights the potential of ctDNA to diagnose recurrence during the post-treatment surveillance period.

These three cases highlight the growing clinical utility of ctDNA in the management of breast cancer, especially in complex or ambiguous situations where tissue biopsy is limited. In each instance, tumor-informed ctDNA enabled early detection of recurrence, guided clinical-decision-making in the absence of confirmatory histology, and helped identify actionable alterations for personalized therapy. In distinguishing between multiple primary malignancies, confirming suspected recurrence without biopsy, or selecting targeted treatment based on the molecular profile, ctDNA served a critical role in the cases in bridging the diagnostic gaps and advancing precision oncology.

As ctDNA technologies continue to mature, their integration into routine cancer care offers the potential for earlier detection of recurrence, more personalized treatment strategies, and improved outcomes.
